# 免疫治疗时代广泛期小细胞肺癌的放疗进展

**DOI:** 10.3779/j.issn.1009-3419.2025.102.20

**Published:** 2025-05-20

**Authors:** Tingting CHEN, Yanling YANG, Haonan HAN, Dongmin LIU, Yajing YUAN, Liming XU

**Affiliations:** ^1^300060 天津，天津医科大学肿瘤医院放疗科，国家恶性肿瘤临床医学研究中心，天津市恶性肿瘤临床医学研究中心，天津市肿瘤防治重点实验室; ^1^Department of Radiation Oncology, Tianjin Medical University Cancer Institute & Hospital, National Clinical Research Center for Cancer, Tianjin's Clinical Research Center for Cancer, Key Laboratory of Cancer Prevention and Therapy, Tianjin 300060, China; ^2^443002 宜昌，肿瘤微环境与免疫治疗湖北省重点实验室，三峡大学基础医学院; ^2^Hubei Key Laboratory of Tumor Microenvironment and Immunotherapy, College of Basic Medical Sciences, China Three Gorges University, Yichang 443002, China; ^3^300060 天津，天津医科大学肿瘤医院麻醉科，国家恶性肿瘤临床医学研究中心，天津市恶性肿瘤临床医学研究中心，天津市肿瘤防治重点实验室; ^3^Department of Anesthesiology, Tianjin Medical University Cancer Institute & Hospital, National Clinical Research Center for Cancer, Tianjin’s Clinical Research Center for Cancer, Tianjin Key laboratory of Cancer Prevention and Therapy, Tianjin 300060, China; ^4^300202 天津，天津市肿瘤医院空港医院放疗科; ^4^Department of Radiotherapy, Tianjin Cancer Hospital Airport Hospital, Tianjin 300202, China

**Keywords:** 肺肿瘤, 广泛期小细胞肺癌, 放疗, 免疫治疗, Lung neoplasms, Extensive-stage small cell lung cancer, Radiotherapy, Immunotherapy

## Abstract

小细胞肺癌（small cell lung cancer, SCLC）是一种胸腔恶性肿瘤，约占肺恶性肿瘤的15%，其被发现时往往就已发生转移。其中广泛期小细胞肺癌（extensive stage-small cell lung cancer, ES-SCLC）约占SCLC总数的2/3。多年以来，由于SCLC对放疗较为敏感，所以放疗在ES-SCLC中占据了重要地位。近年来，免疫检查点抑制剂在ES-SCLC治疗中表现出了较为优秀的抗肿瘤活性，成为ES-SCLC的重要治疗手段之一。然而，在免疫治疗时代，ES-SCLC的放疗将有什么改进?本文将对ES-SCLC的放疗、免疫治疗及综合治疗的临床进展进行综述。

## Abstract (en)

Small cell lung cancer (SCLC) is the thoracic malignant tumor and accounts for about 15% of lung malignancies and transfer often occurs by the time of diagnosis. Extensive stage-small cell lung cancer (ES-SCLC) accounts for about 2/3 of all SCLC. For many years, radiotherapy has occupied an important position in the treatment of SCLC, especially in the treatment of ES-SCLC, because SCLC is more sensitive to radiotherapy. However, in recent years, immune checkpoint inhibitor has shown more excellent antitumor activity in the treatment of ES-SCLC and become the mainstream argument for the treatment of ES-SCLC. However, will radiotherapy be buried by the times among the therapeutic approaches for ES-SCLC? In this article, we will review the clinical progress of radiotherapy, immunotherapy and combination therapy for ES-SCLC.

肺癌是癌症相关死亡的重要原因，同时也是全球最常见的恶性肿瘤之一。小细胞肺癌（small cell lung cancer, SCLC）起源于支气管黏膜上皮细胞，占所有肺癌患者的10%-15%，2015年世界卫生组织（World Health Organization, WHO）将SCLC归类为神经内分泌恶性肿瘤^[1]^，其具有高度侵袭性，预后较差。SCLC分为局限期SCLC（limited stage-SCLC, LS-SCLC）与广泛期SCLC（extensive stage-SCLC, ES-SCLC），后者约占总数的2/3^[2]^。近几十年来，SCLC的治疗方案研究未取得显著进展，ES-SCLC的标准治疗依然为以铂类药物联合依托泊苷为主的化疗^[3]^。虽然大多数SCLC患者对含铂化疗初始反应明显，但几乎所有患者都会有复发和广泛耐药出现^[4]^。近年来，免疫治疗的飞速发展打破了原有的治疗模式，使SCLC治疗有了新的进展，目前ES-SCLC一线治疗方案为免疫治疗联合化疗，但疗效仍然有限。而放疗可以改善肿瘤微环境，与免疫治疗联合应用可促进免疫活化状态，实现更好的抗肿瘤作用，因此，当前许多研究正在探索放疗与免疫治疗的联合应用，以改善ES-SCLC的局部控制以及总生存期（overall survival, OS）。本文主要对目前ES-SCLC的免疫治疗新局面及在免疫治疗时代下的放疗进展进行综述与展望。

## 1 SCLC的病理学特征

SCLC肿瘤细胞呈梭形、圆形或卵圆形，细胞核染色质呈细颗粒状分布，核仁无或不明显，胞质少或裸核，界限不清，常见巢状、小梁状和周边栅栏状生长模式，通常表达如CD56、突触素（Syn）的神经内分泌标志物^[5]^，Ki-67指数通常在50%-100%之间。组织标本中常见坏死、凋亡碎片，间质纤维化和肿瘤浸润淋巴细胞（tumor infiltrating lymphocytes, TILs）。TILs>30%被认为是SCLC独立的负性预后因素之一^[6]^。

## 2 临床特征

SCLC具有早期转移和快速进展等特点。ES-SCLC患者常见的临床表现有咳嗽、呼吸困难、体重减轻和咯血等。常见的转移部位包括对侧肺、肝脏、肾上腺、骨骼、骨髓和脑。有研究^[7]^发现，在初次影像学检查中约40% SCLC患者已发生脑转移。SCLC患者的预后普遍较差，5年生存率不足7%^[4]^。

Gay等^[8]^的研究发现了4种不同的SCLC亚型，主要取决于癌症转录因子的差异表达，如ASCL1、NEUROD1和POU2F3，或转录因子的低表达伴随“炎症”基因的上调，SCLC亚类型分别为SCLC-I、SCLC-A、SCLC-N和SCLC-P。SCLC-N对极光激酶最敏感，SCLC-A对BCL2最敏感，SCLC-P对聚腺苷二磷酸核糖聚合酶（poly ADP-ribose polymerase, PARP）抑制剂最敏感。有意思的是，免疫检查点抑制剂（immune checkpoint inhibitors, ICIs）对SCLC-I的影响最大。尽管相关研究取得了一些小的进展，但在最近的研究中仍存在争议。CASPIAN研究^[9]^证实SCLC-I亚型患者接受免疫化疗（Durvalumab±Tremelimumab+依托泊苷加顺铂或卡铂治疗）后OS最长，KEYNOTE-604研究^[10]^则认为分子亚型与预后无关。

## 3 ES-SCLC的免疫治疗

近几十年来SCLC的标准治疗方案仍是铂类药物联合依托泊苷化疗^[3]^。然而自从进入21世纪的第二个十年，形势发生了一系列的变化，免疫治疗迅速发展，为SCLC带来了新的希望。

### 3.1 ICIs在ES-SCLC一线治疗中的作用

多项关键临床试验^[11-15]^证实，在ES-SCLC一线治疗中，将程序性细胞死亡受体1（programmed cell death receptor 1, PD-1）/程序性细胞死亡配体1（programmed cell death ligand 1, PD-L1）抑制剂与含铂化疗联合应用可以改善患者的OS和无进展生存期（progression-free survival, PFS）。IMpower133试验^[11]^中患者分别接受阿替利珠单抗或安慰剂联合依托泊苷加卡铂的化疗方案，结果提示阿替利珠单抗组相比安慰剂组中位OS有明显改善（12.3 *vs* 10.3个月，HR=0.70，95%CI：0.54-0.91，P=0.007）。对比安慰剂组，阿替利珠单抗组患者的中位PFS也有明显改善（5.2 *vs* 4.3个月，HR=0.77，95%CI：0.62-0.96，P=0.02），说明免疫治疗可提高ES-SCLC患者的生存获益，这项研究正式将免疫治疗带入ES-SCLC的治疗中。另一项CASPIAN研究^[12]^对比了度伐利尤单抗联合依托泊苷加铂类药物4个疗程与接受6个疗程的铂类治疗的疗效。在中位随访14.2个月时，与单独化疗组相比，度伐利尤单抗组中位OS（主要终点）显著改善（13.0 *vs* 10.3个月，HR=0.73，95%CI：0.59-0.91，P=0.005）。不同ICIs联合化疗可明显延长ES-SCLC患者的OS，CAPSTONE-1研究^[13]^结果显示阿得贝利单抗组中位OS为15.3个月，优于安慰剂联合化疗组的12.8个月。EXTENTORCH研究^[14]^显示特瑞普利单抗联合化疗组中位OS为14.6个月，优于安慰剂联合化疗组的13.3个月。同时RATIONALE-312研究^[15]^显示替雷利珠单抗联合化疗组中位OS显著延长。

有研究^[12,13,16]^表明PD-1/PD-L1抑制剂联合化疗的安全性与单纯化疗相当，两组间3-4级不良事件发生率无显著差异。IMpower133研究^[16]^和CASPIAN研究^[12]^表明，阿替利珠单抗组、度伐利尤单抗组与安慰剂组的3-4级不良事件发生率相似。CAPSTONE-1研究^[13]^显示，最常见的治疗相关3-4级不良事件为中性粒细胞计数减少[其中阿得贝利单抗组174例（76%），安慰剂组175例（75%）]、白细胞计数减少[106例（46%）vs 88例（38%）]、血小板计数减少[88例（38%）vs 8例（34%）]和贫血[64例（28%）vs 66例（28%）]。

### 3.2 ICIs在ES-SCLC后线治疗和维持治疗中的作用

ICIs在ES-SCLC后线治疗研究中也显示出一定疗效但存在一定争议。CheckMate 032研究^[17]^发现，在复发性ES-SCLC患者中，纳武利尤单抗单药的客观缓解率（objective response rate, ORR）为11.9%，中位缓解持续时间（duration of response, DoR）达17.9个月。以此为依据，美国食品药品监督管理局（Food and Drug Administration, FDA）曾批准纳武利尤单抗用于既往接受过含铂方案化疗及至少一种其他疗法后进展的转移性SCLC。CheckMate 451试验^[18]^评估纳武利尤单抗作为一线治疗后维持治疗的疗效，结果未能改善OS，导致纳武利尤单抗在美国的SCLC适应证被撤回。III期CheckMate 331试验^[19]^评估纳武利尤单抗作为二线治疗的疗效，也未能得出改善OS的结果。在这项试验中，569例LS-SCLC或ES-SCLC患者在一线铂类为基础的化疗后出现进展，随机（1:1）接受纳武利尤单抗治疗（每2周240 mg）或二线化疗（拓扑替康或氨柔比星），直到出现疾病进展或不可接受的毒性。研究的主要终点是OS，而两组在OS方面没有观察到显著差异（ICIs组 *vs* 化疗组：7.5 *vs* 8.4个月，HR=0.86，95%CI：0.72-1.04，P=0.11）。ICIs组ORR为13.7%，化疗组为16.5%（OR=0.80, 95%CI: 0.50-1.27）。II期IFCT-1603试验^[20]^对阿替利珠单抗的二线治疗进行研究，73例SCLC患者在一线铂类为基础化疗后出现进展，随机（2:1）接受抗PD-L1（每3周1200 mg）或化疗（根据研究者的决定，再次诱导一线化疗或拓扑替康）。主要终点是6周时的ORR。得到的结果不尽如人意：ICIs组ORR为2.3%，化疗组为10%。ICIs组和化疗组的中位OS相似，分别为9.5和8.7个月（HR=0.84, P=0.60）。最近一项回顾性研究^[21]^也认为在标准一线免疫化疗后继续使用免疫治疗不能为ES-SCLC患者带来生存获益。因此，其最佳二线疗法尚未达成共识，复发性ES-SCLC患者的二线治疗选择是非常有限的。

ES-SCLC患者的三线及以上治疗方案仍然有限且存在争议，但免疫治疗取得了一定效果。对两项有关帕博利珠单抗（每3周200 mg或每2周10 mg/kg，直到疾病进展、不可接受的毒性或长达2年）的临床试验（Ib期KEYNOTE-028和2期KEYNOTE-158）进行了汇总^[22]^分析，作为SCLC患者的三线或后续治疗，ICIs取得了显著的效果：ORR为19.3%，超过一半的患者（61%）表现出持久的疗效（≥18个月）。

## 4 ES-SCLC的放疗

SCLC虽然恶性程度高且极易发生转移，但是其对放化疗非常敏感，同时尽管免疫治疗取得一定进展，但获益程度有限，故而放疗对于LS-SCLC亦或ES-SCLC均起到了重要作用。近年来，一项关键研究（CREST）^[23]^结果显示，放疗可改善LS-SCLC患者的OS。但放疗在ES-SCLC患者中的应用，如脑预防照射（prophylactic cranial irradiation, PCI）和胸部巩固放疗（thoracic consolation radiotherapy, TRT）等，效果仍未明确。

### 4.1 ES-SCLC的TRT

多项研究^[23-26]^支持TRT在ES-SCLC中的获益。Jeremic等^[24]^开展的随机对照试验（randomized control trial, RCT）证实，ES-SCLC患者尤其是对于化疗获益较好的患者，接受放疗效果依然很好。此外，Slotman等^[23]^开展的一项III期RCT中，将荷兰等国家共计42所医院总数达498例ES-SCLC患者随机分配到TRT组和对照组中，1年OS率无明显差异（33% *vs* 28%），但是二次分析中放疗组2年OS率取得明显获益（13% *vs* 3%），有胸腔病灶残留的患者TRT获益显著大于无胸腔残余病灶的患者，有胸腔病灶残留的ES-SCLC患者接受放疗OS得到了明显的改善，故可得出结论：对于化疗有效的ES-SCLC患者除进行PCI以外，还应考虑TRT。随后，Gore等^[25]^开展的一项II期RCT评估了诱导化疗后的TRT效果，结论提示TRT组OS无显著改善，但是延缓了进展。近年来，也有系统评价^[26]^分析显示，对于ES-SCLC患者，TRT可明显改善PFS、OS和局部无复发生存期（locoregional recurrence-free survival, LRFS），TRT可使ES-SCLC患者的OS获益（HR=0.65）。所以化疗诱导后行TRT是有获益的。

随着ICIs在ES-SCLC一线治疗中的应用，TRT联合免疫治疗成为研究热点。一些早期研究探索了联合方案的安全性。*Meta*分析^[27]^显示，化疗、免疫治疗联合TRT可显著改善ES-SCLC患者的OS（HR=0.52），且不良反应尚可控，但缺乏前瞻性研究。近年来评价IV期SCLC一线化疗和免疫治疗的试验没有设计放疗的实施^[11,12]^。考虑到肺炎增加的风险，在免疫治疗时代，尚不能明确TRT的参与价值。目前，一项III期临床试验^[28]^正在评估TRT是否增加化疗联合免疫治疗后ES-SCLC患者的获益，仍需更多前瞻性临床试验进一步评估疗效和安全性。不论总人群还是接受维持治疗人群亚组，一线治疗期间是否接受放疗都是除治疗方案外影响PFS的独立预后因素，并且IMpower133亚组分析中复发情况显示以局部复发为主，这均提示我们TRT仍然在化疗联合免疫治疗中有非常重要的地位。

ES-SCLC的TRT剂量多为30-60 Gy，1.5-3 Gy/次。但TRT在ES-SCLC中高剂量与低剂量TRT的效果对比方面，仍存在争议。在生存获益方面，研究^[29]^表明，高剂量TRT可能为ES-SCLC患者带来更好的生存获益。Yoon等^[30]^的一项回顾性研究纳入了85例接受TRT的ES-SCLC患者，比较生物有效剂量（biologically effective dose, BED）>50 Gy与≤50 Gy的效果，结果显示，BED>50 Gy组OS率（40.8% *vs* 12.5%, P=0.006）、PFS率（15.9% *vs* 9.6%, P=0.004）和胸内PFS率（intrathoracic PFS, IT-PFS）（39.3% *vs* 20.5%, P=0.004）均显著优于BED≤50 Gy组。多变量分析进一步确认，BED>50 Gy是OS（HR=0.502, 95%CI: 0.287-0.876, P=0.015）、PFS（HR=0.453, 95%CI: 0.265-0.773, P=0.004）和IT-PFS（HR=0.331, 95%CI: 0.171-0.641, P=0.001）的独立预后因素。另一项回顾性研究^[31]^分析了216例ES-SCLC患者的数据，进一步支持了高剂量放疗的优势，根据TRT剂量分为4组。结果显示，高剂量组（49.5-60.6 Gy）的中位OS（17.5 *vs* 10.9个月，P=0.045）和中位PFS（10.7 *vs* 7.4个月，P=0.014）显著优于低剂量组（≤40.2 Gy）。在Xu等^[32]^的纳入306例ES-SCLC患者的研究中，高剂量组（>50 Gy）和低剂量组（≤50 Gy）的2年OS率分别为32.3%和17.0%（P<0.001），这同样说明高剂量TRT优于低剂量。但也有研究对高剂量TRT的获益提出质疑。CREST试验^[23]^纳入498例ES-SCLC患者，比较了30 Gy/10次TRT与不接受TRT的效果。结果显示，两组1年OS率无显著差异（TRT组 *vs* 对照组：33% *vs* 28%，P=0.066），但TRT组在2年OS率（13% *vs* 3%, P=0.004）和6个月PFS率（24% *vs* 7%, P=0.001）方面体现出优势。在安全性方面，研究结果也存在一定差异。Grønberg等^[33]^的一项纳入170例ES-SCLC患者的研究比较了60和45 Gy的TRT效果，结果显示两组3-4级食管炎和中性粒细胞减少症的发生率相似，且均未有更多的治疗相关死亡事件发生。Yu等^[34]^的RCT在224例LS-SCLC患者中比较了54和45 Gy的TRT效果，研究发现，两组3-4级不良事件（主要包括中性粒细胞减少症、感染、血小板减少症、贫血和食管炎）发生率无显著差异。然而Li等^[26]^的系统评价和荟萃分析发现不良反应发生率存在差异，高剂量TRT组的≥3级食管毒性发生率为4.6%，而低剂量组为0%（P=0.0001）；≥3级支气管肺毒性发生率分别为2.9%和0.8%（P=0.02）。

在分割模式的探究中同样存在争议，Luan等^[35]^的回顾性研究发现，45 Gy分30次（2次/d）组和60 Gy分30次（1次/d）组的PFS分别为11和9个月（P=0.043），但OS并无显著差异。但Han等^[36]^研究则发现，相较60 Gy分30次（1次/d）组，45 Gy分30次（2次/d）组取得了更好的OS和PFS改善。

虽然上述多项研究得出高剂量TRT与更好的生存及局部控制相关，但在ICIs时代现有研究主要集中在放疗与免疫治疗的联合应用。II期LEAD研究^[37]^显示在CASPIAN模式基础上联合低剂量放疗在ES-SCLC一线治疗中能够延长中位PFS，且耐受性良好。其进一步的前瞻性II期临床试验（MATCH试验）^[38]^探究了低剂量放疗与ICIs联合治疗ES-SCLC的效果。研究采用特定剂量的低剂量放疗（15 Gy/5次）与ICIs联合使用，结果显示确认的ORR为87.5%，中位PFS为6.9个月。这一结果提示，低剂量放疗与免疫治疗联合可能产生显著的临床获益。目前尚缺乏直接比较高剂量与低剂量放疗在ES-SCLC免疫治疗中效果的高质量RCT，需要设计严谨的临床试验来评估不同剂量放疗在免疫治疗背景下的疗效和安全性。放疗联合免疫治疗时也应更加谨慎，以避免发生严重不良反应。

在免疫治疗时代的当下，我们仍未清楚哪些ES-SCLC患者最为适合接受TRT，Xu等^[39]^的回顾性分析认为TRT可使寡转移或多转移ES-SCLC患者的OS、PFS和局部控制改善，尤其是寡转移患者获益更多。CREST试验的二次分析^[40]^发现无肝、骨转移和有2个或更少转移的接受TRT的患者的OS和PFS显著提高。提示我们肿瘤负荷较低的患者可能更适合接受TRT。目前也没有可靠的生物标志物对于联合治疗的疗效进行预测，未来需要进一步明确TRT的适应证，探索可能的生物标志物。

### 4.2 ES-SCLC的PCI PCI在LS-SCLC方面有明确证据支持^[41,42]^。

对于ES-SCLC，PCI的获益仍存在争议。ES-SCLC的脑转移很常见，诊断时即有约20%的患者发生脑转移，而2年时发现脑转移比例可达到80%，为了进一步预防脑转移的发生，可以行PCI，但其获益尚不明确。Soon等^[43]^对2003至2010年在新加坡癌症中心治疗的符合条件的223例无脑转移ES-SCLC患者进行的一项试验表明，对比2003至2006年和2007至2010年PCI使用有所增加（32% *vs* 10%, P=0.044）；而且与无PCI相比，PCI可改善OS（HR=0.22, 95%CI: 0.10-0.47, P<0.001）。2007年以来，在ES-SCLC人群中PCI应用有所增加，证实了对于ES-SCLC患者，PCI是有获益的，PCI有助于ES-SCLC患者的生存率提高。而在另一项Slotman等^[41]^发起的EORTC的随机研究中，对化疗有效的ES-SCLC患者分别接受5-12次20-30 Gy PCI或者不接受PCI治疗，主要终点为出现症状性脑转移的时间，结果显示，接受PCI治疗的患者组患者有症状的脑转移风险较低（HR=0.27, 95%CI: 0.16-0.44, P<0.001）；在次要终点OS方面，与对照组相比，PCI组也有所改善（6.7 *vs* 5.4个月，HR=0.68，95%CI：0.52-0.88，P=0.0003），所以该研究为PCI作为对一线化疗敏感的ES-SCLC患者的治疗标准奠定了基础。但日本的一项研究^[44]^表示，充分的磁共振成像（magnetic resonance imaging, MRI）评估确定脑转移治疗后与无脑转移PCI治疗的生存率没有明显差异。正在进行的SWOG S1827/MAVERICK试验^[45]^将进一步评估PCI治疗与进行MRI监测的生存获益，评估是否可以用脑部定期复查取代PCI。

目前研究者还普遍关注的问题就是PCI的神经毒性风险。PCI可能会导致神经认知功能障碍等不良反应，影响患者的生活质量。RTOG 0212试验^[46]^发现，>60岁患者中约83%在接受PCI治疗12个月后出现慢性神经毒性，有研究^[47]^认为，新技术应用后，PCI的神经毒性风险可能被其获益所抵消。负责记忆和高级神经认知功能的结构主要存在于海马区域。前瞻性单臂II期研究（RTOG 0933）^[48]^发现，WBRT期间对患者海马体进行适形保护，经Hopkins词语学习测验修订版（Hopkins verbal learning test-revised, HVLT-R）和生活质量（quality of life, QOL）评估后证实患者记忆损伤降低。但NCT01780675研究^[49]^并未发现接受海马保护PCI术的SCLC患者的认知能力的下降的概率低于接受标准PCI，海马保护的有效性仍需更大规模的研究来证实。同时临床前研究已证明几种物质对神经保护有积极作用。RTOG0614研究^[50]^将508例符合条件的患者进行随机分配，在开始放疗后3 d内随机接受安慰剂或美金刚（20 mg/d）治疗，持续24周。随后对患者进行认知功能的系列标准化测试，结果显示，随着时间的增加，美金刚组患者的认知功能更好。目前仍有许多药物相关研究正在进行，未来药物神经保护也可能作为避免PCI神经毒性的重要策略。

对于有脑转移症状且没有立即进行系统治疗指征的SCLC脑转移患者，建议在系统治疗前进行颅内放疗，对于无脑转移症状的患者则可以在全身系统治疗后进行颅内放疗。在同步治疗方面，现有临床证据尚未充分确立同步治疗策略。一项LS-SCLC免疫治疗（帕博利珠单抗21 d内给药）同步PCI的I/II期试验^[51]^发现，与未接受PCI的患者相比，接受ICIs同步PCI患者的中位OS明显延长（39.5 *vs* 30.0个月，P<0.05），而在非同步进行PCI与免疫治疗联合应用的临床试验中，多数研究未呈现显著阳性结果。有关联合治疗毒性方面，NSCLC脑转移患者联合治疗耐受性良好。NSCLC脑转移放疗联合ICIs最常见的神经系统不良反应是发热、疲劳、恶心和癫痫发作，主要为1-2级，很少出现3-4级，无5级不良反应发生。SCLC脑转移患者应用放疗联合ICIs治疗的疗效尚未明确。目前SCLC脑转移患者治疗大多依赖于IMpower133^[11]^、CASPIAN^[12]^、KEYNOTE-604^[52]^等经典试验的亚组分析，联合免疫治疗不会对伴有脑转移SCLC患者产生更严重的毒性。回顾性研究^[53]^也表明，与PCI/WBRT相比，PCI/WBRT+阿替利珠单抗不会增加神经毒性的发生风险。IMpower133研究^[11]^允许患者在同步免疫化疗治疗完成后的维持阶段进行PCI，且在每组中接受PCI患者比例均为11%。KEYNOTE-604研究^[52]^中，在第4个周期后达到完全缓解（complete response, CR）或部分缓解（parital response, PR）的患者可以根据研究人员的判断进行评估是否接受PCI治疗，最终帕博利珠单抗+EP组有27例患者（11.8%）接受了PCI，安慰剂+EP组则有32例患者（14.2%）。IMpower133^[11]^和KEYNOTE-604^[52]^试验均未发现接受PCI联合免疫治疗的患者存在安全问题。该临床方案目前尚无大型前瞻性临床研究，仍需更多研究来权衡利弊。放疗作为ES-SCLC多年来一直沿用的基本治疗手段，经历了历史的检验，但联合免疫治疗仍需要从多角度论证其适应证。例如先进的放疗技术如调强放射治疗（intensity-modulated radiation therapy, IMRT）、容积旋转调强放疗（volume modulated arc therapy, VMAT）、影像引导放疗和立体定向放疗有望更精确地实施放疗，以降低严重不良事件的发生风险，未来需要进一步评估探索这些新技术在SCLC中的应用价值^[54]^。

此外，我们还需要关注特殊人群，如老年、体能状态评分差或有脑转移的SCLC患者。在过去的40年间，SCLC患者中老年患者的比例显著增加^[55]^。老年患者通常意味着体能状态评分较差、身体虚弱，伴有多种合并征，并且比年轻患者更容易遭受严重的治疗相关毒性^[56]^，因此需要进一步研究来制定个体化的治疗策略。Takahashi等^[44]^的研究中PCI组和观察组中分别有约47%和46%的患者年龄≥70岁，但PCI并未使患者获得更长的OS。该研究得出由于存在认知功能下降的风险且老年患者的风险更高，对特定ES-SCLC患者应仔细考虑是否应用PCI治疗。而另一项针对ES-SCLC患者的NCT00016211试验^[41]^认为PCI可降低有症状脑转移的发生率，并延长患者的无病生存期（disease-free survival, DFS）和OS。在已经得到结果的研究中，老年患者的代表性不足，且PCI的数据不一致，我们应根据患者的疾病分期、对初始治疗的反应、体能状态以及合并症的情况，严格评估谨慎选择PCI的应用。Schild等^[57]^的研究发现超高龄患者（>80岁）虽然伴有合并症且功能储备有限，但他们似乎与年轻人一样受益于更积极的治疗且在生存方面受益，因此更应根据每位患者的愿望和需求进行个体化治疗。在ES-SCLC的治疗逐渐被化疗和免疫主导的大背景的改变之下，探寻更多放疗的获益，寻找更加适合的出路，是我们应该重视的问题。

## 5 对于ES-SCLC治疗方式的对比与讨论

### 5.1 化疗与放疗结合前景

半个世纪以来，由于SCLC患者对于放疗和化疗敏感，放疗和化疗一直以来都是SCLC尤其是ES-SCLC的重要治疗手段，许多患者从中受益。但是首次治疗效果虽好，但是其具有易复发、易耐药、二三线治疗效果差等特点，导致ES-SCLC的治疗在宏观效果上停滞不前。

### 5.2 免疫治疗与放疗结合前景

肿瘤免疫化疗可改善ES-SCLC患者的OS，已成为ES-SCLC的新标准一线治疗方案，迎来了免疫治疗的新时代。许多人认为，进入了免疫治疗时代，过去的一系列放疗手段或许已经过时，但是事实真的如此吗?虽然在免疫时代的当下，放疗的获益比并非拔尖，其副作用和对患者机体的损害也应受到重视，但是，其在国内治疗的受众及治疗范围广泛，而且其门槛相对较低，在目前国内环境及医疗条件下，放疗为更多的患者提供了生存的可能性。免疫治疗方案也并非尽善尽美，虽然ICIs联合化疗在ES-SCLC中取得了一定疗效，但与在其他实体瘤中取得的疗效相比，其获益幅度仍然有限。目前仍缺乏有效的预测生物标志物，并且几乎所有SCLC患者都会出现耐药现象，这也是重要问题。如同上文所述，我们不难推测，虽然免疫治疗时代其光芒一定程度上掩盖了传统放化疗或手术方案，但是对于整个ES-SCLC治疗体系而言，其不仅不能完全替代其他治疗方案，同时也为其他如手术、化疗、放疗等治疗手段提供了新的视野和思考方式。近年来，放疗联合免疫治疗备受瞩目，它们之间相辅相成的作用已经在非小细胞肺癌^[58]^、黑色素瘤^[59]^、肝内胆管癌^[60]^的诊疗中取得了一些成效。放疗（尤其是低剂量放疗，具有较低的免疫抑制和辐射毒性）与免疫治疗联合应用在SCLC中同样显示出巨大潜力。放疗联合免疫治疗可以提高肿瘤缓解率，减缓疾病进展，而同时远隔反应也可能潜在地延缓疾病进展^[61]^。近年来，许多临床试验关注将放疗（包括低剂量放疗）加入ES-SCLC的一线治疗，以期获得协同增效作用，但具体疗效和安全性有待进一步评估。TRT与免疫肿瘤化疗联合应用的获益，目前尚缺乏前瞻性随机对照研究的证据^[62]^。JCOG2002研究^[28]^正在评估免疫肿瘤化疗后是否需要额外进行TRT。PCI在ES-SCLC中的作用也仍需进一步研究确定。MAVERICK临床试验（NCT04155034）正在评估PCI在免疫治疗时代下的获益^[45]^。有研究^[63]^表明，化疗和放疗可以通过增加肿瘤细胞的初始免疫性、增加主要组织相容性复合体I（major histocompatibility complex I, MHC-I）表达或直接激活免疫效应，促进免疫反应。有证据^[64]^表明，放疗或化疗可以通过减少肿瘤的体积来缓解肿瘤的免疫抑制，从而建立起对于T细胞激活更加适应的稳态场景，因此，从这方面思考，免疫治疗的有效性可以通过放化疗的联合铺垫来增强。未来我们可以开展更多大规模、前瞻性的临床试验，全面评估放疗联合免疫治疗应用于ES-SCLC的疗效和安全性，探索两者的最佳时序、放疗剂量和技术等^[28]^。通过基础研究深入探究放疗增强免疫治疗疗效的分子机制，如肿瘤抗原呈递、免疫原性细胞死亡、免疫微环境重塑等，争取为优化联合治疗策略提供更多理论依据。探索其他免疫治疗策略（如PARP抑制剂、抗体偶联药物等）与放疗联合应用于SCLC的可能性，能否将ES-SCLC应用放疗联合免疫治疗的经验应用在其他恶性肿瘤之上，这些都是我们未来可以探索的方向。

当前来看，还有许多正在开展或者结束的项目，也在探寻着多种方案联合治疗SCLC的效果（表1^[65-76]^），或许在将来，我们突破了视野和角度的限制，放疗或其他传统治疗方案，也能在针对ES-SCLC或者其他恶性肿瘤的治疗上有新的切入点和领导领域，并重新发挥出新的作用。

**表 1:** 针对多种联合治疗方案治疗SCLC的效果的研究汇总

Trial code	Intervention	Trial phase	Inclusion criteria	n	Endpoint
Immunotherapy+Chemotherapy					
NCT03963414^[65]^	Durvalumab+Tremelimumab+Platinum-Etoposide *vs* Durvalumab+Platinum-Etoposide	I	Untreated ES-SCLC	18	Safety and tolerability
NCT03568097^[66]^	Avelumab+Platinum-Etoposide	II	Advanced SCLC	55	Primary: 1-yr PFS; Secondary: OS, BOR, ORR
NCT03382561^[67]^	Nivolumab+Platinum-Etoposide vs Platinum-Etoposide	II	ES-SCLC	160	Primary: PFS; Secondary: OS, BOR, AEs
NCT04028050^[68]^	Atezolizumab+Platinum-Etoposide	IIIB	Untreated ES-SCLC	155	Primary: Safety; Secondary: OS, PFS, ORR, DoR
Immunotherapy+Radiotherapy					
NCT03043599^[69]^	Ipilimumab+Nivolumab+Thoracic radiation therapy	I/II	Post-chemotherapy ES-SCLC	21	Primary: (1) recommended phase II dose; (2) PFS; Secondary: OS
NCT03262454^[70]^	Atezolizumab+Continuous hypofractionated radiotherapy	II	Relapsed SCLC	35	Primary: ORR; Secondary: PFS
NCT02701400^[71]^	Tremelimumab+Durvalumab vs Tremelimumab+Durvalumab+SBRT/HRT	II	Relapsed SCLC	18	Primary: PFS, ORR; Secondary: OS, irORR
Immunotherapy+Chemotherapy+Radiotherapy					
NCT02402920^[72]^	LS-SCLC: Pembrolizumab+Chemotherapy +Radiotherapy ES-SCLC: Pembrolizumab+Radiotherapy	I	LS-SCLC, ES-SCLC	83	Primary: AEs, optimal dose; Secondary: ORR, PFS, OS, BR
NCT03540420^[73]^	Atezolizumab following CRT *vs* CRT alone	II	LS-SCLC	212	Primary: 2-yr survival; Secondary: PFS, BORR, AEs
NCT03585998^[74]^	CRT+Durvalumab followed by consolidation Durvalumab	II	Post-chemoradiotherapy LS-SCLC	51	Primary: PFS; Secondary: OS, safety
NCT02046733^[75]^	Nivolumab+Ipilimumab *vs* No intervention	II	Post-chemoradiotherapy LS-SCLC	222	Primary: PFS; Secondary: OS, BORR, AEs
NCT03811002^[76]^	Atezolizumab+Platinum-based chemotherapy (Etoposide)+3D-CRT or IMRT *vs* Platinum-based chemotherapy (Etoposide)+3D-CRT or IMRT	III	LS-SCLC	545	Primary: OS; Secondary: PFS, AEs, BORR, etc

LS-SCLC: limited stage-small cell lung cancer; ES-SCLC: extensive stage-small cell lung cancer; PFS: progression-free survival; OS: overall survival; BOR: best overall response; BORR: best overall response rate; ORR: objective response rate; AEs: adverse events; DoR: duration of response; irORR: immune-related objective response rate; BR: biomarker response; CRT: concurrent chemoradiotherapy; HRT: hypofractionated radiation therapy; SBRT: stereotactic body radiation therapy; IMRT: intensity-modulated radiation therapy.

## 6 结语

目前来说，在治疗ES-SCLC方面，化疗仍是其他治疗的基础，在此基础上，更准确地联合手术、放疗、靶向治疗、免疫治疗，是针对ES-SCLC患者治疗的重要方法。虽然近年来免疫治疗技术突飞猛进，逐渐成为治疗ES-SCLC的一线标准，但是经历过历史考验和完善的放疗及其他治疗方式也不会黯然退出历史舞台。诚然，对于SCLC患者，尤其是ES-SCLC患者来说，当前的治疗现状还不够令人满意，各种方案的疗效也差强人意，我们仍然需要努力寻求未来的治疗模式，同时也仍要继续综合计算各项治疗单独和联合治疗的收益，融合多种治疗方案综合探寻，或许在未来不懈的努力下，SCLC的研究将会出现新的成果。总之，免疫治疗为ES-SCLC带来了新的曙光，但放疗在ES-SCLC治疗中仍扮演着重要角色。未来仍有诸多值得深入探讨的领域，需要临床医生和科研工作者的共同努力，以进一步改善这一疾病的治疗现状。
